# A Simulation Environment for Benchmarking Sensor Fusion-Based Pose Estimators

**DOI:** 10.3390/s151229903

**Published:** 2015-12-19

**Authors:** Gabriele Ligorio, Angelo Maria Sabatini

**Affiliations:** The BioRobotics Institute, Scuola Superiore Sant’Anna, Piazza Martiri della Libertà 33, Pisa 56125, Italy; angelo.sabatini@sssup.it

**Keywords:** simulation, sensor modeling, sensor fusion, performance evaluation

## Abstract

In-depth analysis and performance evaluation of sensor fusion-based estimators may be critical when performed using real-world sensor data. For this reason, simulation is widely recognized as one of the most powerful tools for algorithm benchmarking. In this paper, we present a simulation framework suitable for assessing the performance of sensor fusion-based pose estimators. The systems used for implementing the framework were magnetic/inertial measurement units (MIMUs) and a camera, although the addition of further sensing modalities is straightforward. Typical nuisance factors were also included for each sensor. The proposed simulation environment was validated using real-life sensor data employed for motion tracking. The higher mismatch between real and simulated sensors was about 5% of the measured quantity (for the camera simulation), whereas a lower correlation was found for an axis of the gyroscope (0.90). In addition, a real benchmarking example of an extended Kalman filter for pose estimation from MIMU and camera data is presented.

## 1. Introduction

Synthetic ground-truth data are widely used for algorithm validation in several applications, including pose (orientation and position) estimation methods [1,2,3,4,5]. The workflow of algorithm validation typically involves the following steps: the design of the algorithm, the assessment of its performance on simulated data and, finally, experimental validation. Although experimental validation is essential to legitimate the conclusions of any scientific investigation, it is well known that several undesired and sometimes uncontrollable factors may affect the experimental results. These factors are related to either the sensors, including those generating ground-truth data (intrinsic factors), or the external environment (extrinsic factors). On the other hand, simulation environments offer the opportunity to control both intrinsic and extrinsic factors, generate accurate ground-truth data and perform replications and statistical analyses.

The work carried out on sensor simulations in a pose estimation context is analyzed below.

An inertial measurement unit (IMU) signal simulator was presented in [6]. The adopted object-oriented language approach based on the C++ language allowed the project to be extensible and modular. Realistic motion trajectories were generated starting from synthetic instances of angular velocity and acceleration signals. These signals were then modified to account for several error sources affecting sensor outputs, e.g., sensitivity and cross-axis sensitivity, bias, misalignments between reference frames, measurement (white) noise and quantization noise. The simulation framework was validated by comparing the generated signals with those from a real IMU.

An IMU signal generator was developed in [7] to gain insight into the operation and performance of pedestrian dead reckoning (PDR) algorithms. In particular, the authors aimed at generating simple and realistic foot motion trajectories in three-dimensional (3D) space, without accounting specifically for any error source apart from the white measurement noise. Thanks to the use of the IMU signal generator, some critical points were discovered in the PDR algorithm, leading to a new design with better performances.

An object-oriented language approach was also adopted to implement IMUSim, a Python-based magnetic-inertial measurement unit (MIMU) simulator proposed specifically for applications in the field of human motion analysis [8]. The ground-truth pose was obtained using stereophotogrammetric reference data from an optical motion capture system. The filtered motion trajectories were processed through the rigid body kinematic equations to obtain the angular velocity and the linear acceleration of the simulated IMU. Models of the measurement noises and biases that affect magneto/inertial sensors were implemented. Additional interesting aspects of IMUSim were the introduction of models describing the Earth’s magnetic field distribution and the simulation of wireless sensor network operation.

In [9], another MIMU simulator was implemented, which modeled the sensor frequency response with a first-order dynamics. In addition, a more complex model for the sensor biases was introduced. Finally, the Earth’s fixed frame, which rotates together with the Earth, was distinguished from the inertial reference frame, which does not rotate with the Earth.

As for the simulation of vision-based systems for, e.g., navigation and ego-motion estimation, the visual measurements of interest typically consist of two-dimensional (2D) discriminative features that can be present in an image, such as corners or lines [10,11]. These features, resolved in a 2D reference frame attached to the image plane, are typically extracted from grayscale images using *ad hoc* algorithms called feature trackers [10,12]. Therefore, from the algorithm designer’s point of view, the expected outputs from a camera simulator are the synthetic 2D points (or lines) returned from a virtual feature tracker. The standard procedure to simulate a vision system involves the creation of ground-truth three-dimensional (3D) points associated with their projection onto the image plane [1,2,13]. Such a projective transformation requires a camera model (e.g., pinhole, fisheye) and the ground-truth camera pose and orientation with respect to the reference frame in which the features are defined. Correspondence between the same features in consecutive images is usually what is needed for camera pose estimation. In real conditions, noise, time-varying lighting conditions, fast movements and occlusions contribute to producing feature mismatches, which represent a major problem in vision-based ego-motion estimation [14]. It would be desirable for a camera simulator to account for this kind of disturbance.

The aim of this work is to present the simulation environment that we developed for six DOF pose estimator benchmarking. The code is attached to this paper as Supplementary Material. This paper therefore represents a useful reference for possible users of the framework here presented. Unlike the works analyzed above, the simulation of different kinds of sensors is supported simultaneously. The suite of sensors includes magnetic/inertial sensors and monocular cameras; however, new sensing modalities can be easily added by inheritance. Several sensors may be instantiated and integrated during a simulation session in order to obtain a sensing pool whose output data can be used to feed a pose estimation algorithm. In this paper, we first describe the simulation environment and the steps taken for its validation against real-life sensor data. Then, we report how IMU and camera data from a real experiment were replicated in a simulation to show the correctness of the synthetic sensor outputs. In addition, an actual benchmarking example concerning a sensor fusion-based extended Kalman filter (EKF) for orientation estimation is presented to show the usefulness of the proposed simulator. Finally, an example of magnetic disturbance simulation is shown, since it represents a useful functionality for replicating a realistic magnetic environment.

## 2. Methods

### 2.1. Simulation Framework

**Figure 1:**
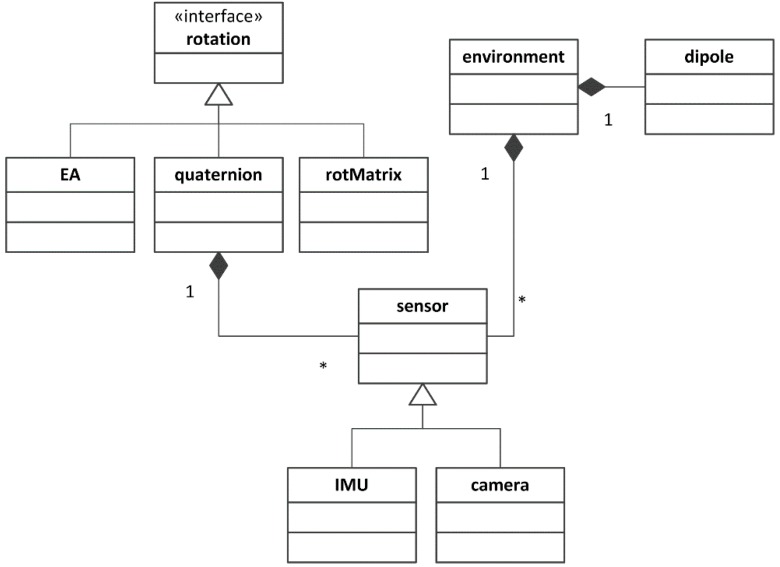
Unified Modeling Language (UML) diagram of the proposed simulation framework.

The modularity and extensibility inherent in an object-oriented programming approach are valuable features when developing a simulation environment [6]. The simulation environment presented in this paper was developed in MATLAB (the MATLAB code is attached as Supplementary Material), exploiting its object-oriented programming features. The Unified Modeling Language (UML) diagram underlying the simulator project is shown in Figure 1. The nodal point of the UML class diagram is represented by the *sensor* class, which contains all the relevant information needed to describe the rigid body motion. Starting from the specification of the body pose, linear acceleration and angular velocity are computed and stored within the *sensor* class. For this purpose, two reference frames are introduced: the Earth-fixed navigation frame {n} and the sensor body frame {b}. Following the notation of this paper, $b^{n}$ is the position of {b} in {n}, whereas $q_{k}^{bn}$ and $R_{k}^{bn}$ denote, respectively, the quaternion and the orientation matrix encoding the rotation from {n} to {b} (the subscript k denotes the *k*-th time sample of the simulation).

The classes named *IMU* and *camera* inherit from the *sensor* class. Each class implements a different measurement model so as to calculate the simulated sensor output from the inherited motion information.

The measurement models may require the specification of some external quantities, e.g., the Earth’s magnetic field (for the magnetic sensors embedded in an MIMU), the gravity vector field (for the accelerometers embedded in an MIMU) and the three-dimensional (3D) scene features (for visual camera systems). Therefore, an *environment* class was created to capture and collect all information of this sort. Each *sensor* gains access to all of the contextual information through the same *environment* instance, as described in Section 2.1.2. All classes were implemented and debugged using the test-driven development (TDD) approach by using the MATLAB unit testing framework presented in [15].

#### 2.1.1. *Rotation* Classes

A class hierarchy was implemented to represent 3D rotations and handle all of the related operations more easily. Three parameterizations of orientation were included: rotation matrix (*rotMatrix* class), quaternion (*quaternion* class) and Euler angles (*EA* class). These classes derive from the *rotation* interface class. The *quaternion* class adopts the conventions proposed in [16].

#### 2.1.2. *Environment* Class

- The *environment* class collects the contextual information of interest to the classes named *IMU* and *camera*. Specifically, the contextual information concerns:
- The expression of the Earth’s magnetic and gravity vector fields resolved in {n};
- The scene, namely the coordinates of the 3D points in {n} that have to be projected onto the image plane {i} (see Section 2.1.6);
- The reference to an array of *dipole* instances (see Section 2.1.3) that are intended to model local magnetic disturbances.

Since the environment is common to all sensors involved in the same simulation/experiment, a singleton pattern [17] was used to model the *environment* class. The user is required to create the *environment* object at the beginning of the simulation and to specify all contextual information. The singleton pattern prevents creating copies of the instance by mistake, which helps keep the information about the environment consistent.

#### 2.1.3. *Dipole* Class

**Figure 2:**
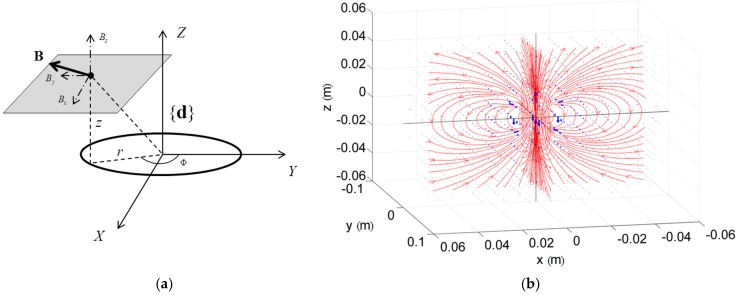
*Dipole* class modeling: (**a**) Dipole reference frame; (**b**) typical spatial distribution of the dipole magnetic field computed by means of Equation (1).

Magnetic disturbances due to nearby hard-iron objects or magnetic sources add to the Earth’s magnetic field. The *dipole* class models the spatial distribution of the field generated by a magnetic source according to the dipole equations presented in [18]. Referring to Figure 2a, {d} is the reference frame attached to the dipole. The magnetic field $B^{d}=(B_{x}^{d},B_{y}^{d},B_{z}^{d})$ generated at the point $p^{d}=(x,y,z)$ can be written as follows:

(1) Bxd=3μ0Mrh4π(r2+h2)52sinΦByd=3μ0Mrh4π(r2+h2)52cosΦBzd=μ0M4π(r2+h2)52(2r2−h2)

where M is the dipole magnetization (A·m^2^), $\mu_{0}$ is the magnetic permeability of the air (N/A^2^) and (r,h,Φ) are the cylindrical coordinates of $p^{d}.$ In addition to the value of the dipole magnetization, the position and orientation of {d} relative to {n} (*i.e.*, $d^{n}$ and $q^{dn})$ may be set by the user when creating a new *dipole* instance. In Figure 2b, a typical magnetic field distribution generated with Equation (1) is depicted.

Suppose that an arbitrary number of dipoles is attached to the *environment* singleton; the magnetic field in a 3D point, resolved in {n}, is the sum of the Earth’s magnetic field (stored in the *environment*) and the contributions from all of the *dipole* instances in that point.

#### 2.1.4. *Sensor* Class

A generic sensor was modeled as an entity whose output may depend on the body pose and its time derivatives. The *sensor* class models the discrete-time kinematics of the rigid body; moreover, it contains a reference to the *environment* object. Therefore, all of the derived classes (*i.e.*, *IMU* and *camera*) are allowed access to the environmental information required for implementing the respective measurement models.

**Figure 3:**
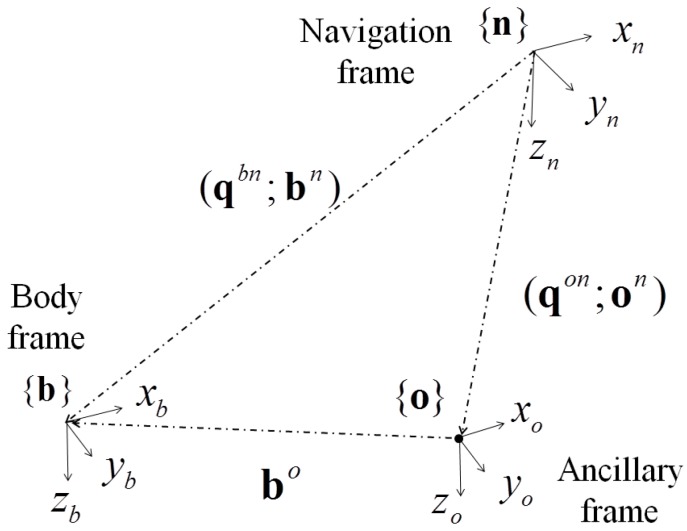
Reference frames considered for the *sensor* class implementation.

In addition to the reference to the *environment* object, $q_{k}^{on}$, $o_{k}^{n}$ and $b^{o}$ are required for constructing a *sensor* instance, where {o} is an ancillary reference frame aligned with {n} (*i.e.*, $q_{k}^{bn}=q_{k}^{on}),$ with a constant lever arm $b^{o}$ with respect to {b} (Figure 3). The sensor position at the *k*-th time instant is calculated as: 

(2) $$b_{k}^{n}=o_{k}^{n}+R_{k}^{no}b_{k}^{o}$$

The body angular velocity $\omega_{k}^{b}$ may be computed as suggested in [16]:

(3) $$\omega_{k}^{b}=2{\dot{q}}_{k}^{bn}⊗q_{k}^{nb}$$

The linear velocity ${\dot{b}}_{k}^{n}$ and acceleration ${\ddot{b}}_{k}^{n}$ are estimated by numerical differentiation. The rationale of expressing $b^{n}$ relative to $o^{n}$ is that the user is allowed to attach the simulated sensor to any point onto the rigid body. For example, suppose that $o^{n}$ and $q_{k}^{bn}$ are known from an optoelectronic motion capture system as in [8]. If no lever arm $b^{o}$ is specified during the *sensor* instance construction, {b} and {o} are assumed to be coincident $(b^{o}={\left[0\;0\;0\right]}^{T}).$ Conversely, by changing the value of the lever arm, the user can move the simulated sensor from $o^{n}$ to any desired point onto the rigid body. The components of centripetal acceleration can be thus taken into account when the second order derivative of $b^{n}$ is computed.

#### 2.1.5. *IMU* Class

The *IMU* class contains three simulated sensors: a tri-axial gyroscope, a tri-axial accelerometer and a tri-axial magnetic sensor. The *IMU* outputs are calculated based on the kinematics inherited from the *sensor* class. Error sources that are typical of magneto/inertial sensors can be user selected. Following the models of [19], the gyroscope and accelerometer measurement models are:

(4) ω^kb=Sg ωkb+bg+vkga^kb=Sa Rkbn(b¨kn−gn)+ba+vka

where ${\hat{\omega }}_{k}^{b}$ and ${\hat{a}}_{k}^{b}$ are the simulated measurements of, respectively, the angular velocity and the linear acceleration, $g^{n}$ is the gravity vector in {n},Sg,Sa are the sensitivity matrices, bg,ba are the biases and vkg,vka are the zero-mean white noises of the simulated gyroscope and the accelerometer, respectively.

For the simulation of the magnetic sensor, we followed the model presented in [20]:

(5) m^kb=SmRkbn(hn+dhn)+bm+vkm

where ${\hat{m}}_{k}^{b}$ is the simulated magnetometer output, $h^{n}$ is the Earth’s magnetic field, $dh^{n}$ accounts for all magnetic perturbations, Sm is the sensitivity matrix, bm is the bias and vkm is the zero-mean white noise of the magnetic sensor. The user is allowed to specify the parameters of the measurement models, including the noise covariance matrices. Scale factor errors, misalignments and cross-axis sensitivities may also be set through the sensitivity matrices. The default value of all sensitivity matrices is the identity matrix. $dh^{n}$ represents the contribution of the *dipole* instances stored within the *environment* sensed by the simulated magnetometer.

As proposed in [9,21], the bias terms in Equations (4) and (5) were simulated as the sum of a constant value, a random walk process and a Gauss–Markov process.

#### 2.1.6. *Camera* Class

Vision-based motion tracking relies on three main steps: (1) image acquisition; (2) two-dimensional (2D) image feature detection and matching; (3) pose estimation. The *camera* class simulates the first two steps by returning the typical output of a feature tracker, namely the 2D correspondences in two consecutive images [12,14].

The *camera* class implements the projective transformation producing the 2D feature positions in {i}. A standard pinhole camera [14] is simulated for undistorted image features pkiu:

(6) pkiu=[fxαccx0fyccy001]Pb=KPb=KRkbn(Pn−bkn)

where $P^{n}$ and $P^{b}$ yield, respectively, the coordinates of the 3D points in the navigation and camera frame, respectively, whereas K is the camera calibration matrix [22]. Intrinsic parameters, *i.e.*, the camera focal $f_{x}$ and $f_{y},$ skew coefficient α, principal point coordinates $cc_{x}$ and $cc_{y}$ and the resolution, are represented in the *camera* instance and can be set as required by the user. The extrinsic camera parameters, *i.e.*, the camera position and orientation with respect to the reference frame in which the 3D features are defined, are inherited from the *sensor* class. Non-linear lens distortion may be simulated by specifying the five distortion coefficients relative to the model proposed in [23]. The 3D points of the scene are available from the *environment*.

At each time-step, the ideal camera measurements consist of the 2D coordinates of all visible image features $\left\{p_{k}^{i}\right\}$ and their correspondences with their counterparts $\left\{p_{k-1}^{i}\right\}$ in the previous image frame. Note, however, that since new (old) features may appear (disappear), the number of visible features in two consecutive frames is variable. Therefore, the number of correspondences is less than (or equal to) the size of the smaller feature set.

Each of the ideal 2D coordinates $p_{k}^{i}$ are then corrupted with zero-mean white noise vkc:

(7) p^ki=pki+vkc

Feature mismatching is one of the typical problems with feature trackers [14]. To simulate this phenomenon, the user is asked to provide two numbers between 0 and 1, which correspond to the percentage of frames in which mismatching takes place and the percentage of wrong correspondences. These numbers are interpreted as the probabilities of mismatch events, which are randomly generated accordingly.

### 2.2. Case Study

To show the usefulness of the proposed simulation framework, a case study is reported in this work. Simulated data from *camera* and *IMU* instances were used to test the consistency of a simple quaternion-based EKF that fuses visual and inertial measurements. The consistency of a state estimator is defined as its capability to converge, asymptotically, towards the true state [24]. The most important consistency check implies that the estimation errors are zero mean with a covariance, which is in accord with the uncertainty estimated by the filter. This check is feasible only when simulated data are considered [24]. The plausibility of the estimated covariance is of primary importance for a Kalman filter, since it involves the calculation of the optimal Kalman gain. If the covariance estimation is wrong, then the Kalman gain is not optimal.

The vector state $x_{k}$ is represented by $q_{k}^{bn}:$

(8) $$x_{k}=q_{k}^{bn}$$

In the prediction step, the state is projected by means of gyroscope measurements ${\hat{\omega }}_{k-1}^{b}.$ If the angular velocity is assumed to be constant during the sampling period *T_s_*, the predicted state quaternion ${\tilde{x}}_{k}^{-}$ can be calculated as follows [25]:

(9) $${\tilde{x}}_{k}^{-}=\exp\left(\left[\begin{array}{cc} \left[{\hat{\omega }}_{k-1}^{b}\times \right] & {\hat{\omega }}_{k-1}^{b}\\ {\left(-{\hat{\omega }}_{k-1}^{b}\right)}^{T} & 0 \end{array}\right]T_{s}\right){\tilde{x}}_{k-1}^{+}$$

where [u×] is the skew-symmetric matrix operator [25] and ${\tilde{x}}_{k-1}^{+}$ is the updated state at the previous time instant.

If the magnetometer and accelerometer are corrupted only by the white measurement noise, the two following nonlinear measurement equations may be used for correcting the prediction error:

(10) m^kb=qkbn⊗hn⊗qknb+vkma^kb=qkbn⊗−gn⊗qknb+vka

As described in [26], Equation (10) must be linearized to be exploited in the EKF framework.

(11) mkb≈∂(qkbn⊗hn⊗qknb)∂qkbn|qkbn=q^kbn−qkbn=Hkmqkbnakb≈∂(qkbn⊗−gn⊗qknb)∂qkbn|qkbn=q^kbn−qkbn=Hkaqkbn

As for the visual block, if at least four 2D-3D correspondences relative to non-collinear points are known, the camera pose may be inferred using the direct linear method (DLT), [14]. Therefore, the DLT-based orientation estimate can be used as an additional measurement channel for the EKF:

(12) q^kbnDLT=qkbn+vkDLT

where q^kbnDLT is the quaternion estimated by the DLT method and vkDLT is the DLT measurement noise.

When designing a visual-inertial-based sensor fusion method, a multi-rate strategy must be adopted to cope with the different sampling periods of the two sensors. In this filter, the EKF measurement model is switched between Equations (11) and (12) according to the kind of current data sample. The prediction step does not change, since the angular velocity is supposed to be constant within the *IMU* sampling period. Note that in the proposed simulation framework each *sensor* instance has its own sampling period, which was set at 100 Hz for the *IMU* and 30 Hz for the *camera*.

### 2.3. Experimental Validation

**Figure 4:**
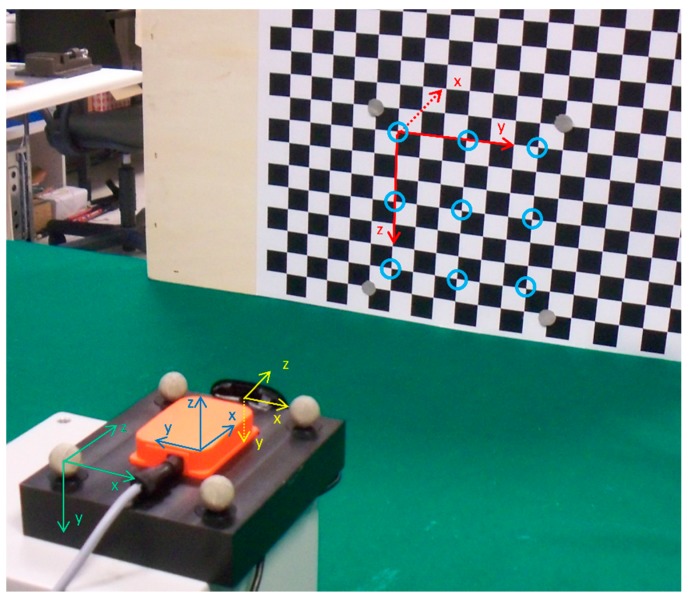
Experimental setup: the camera (yellow), IMU (blue), local Vicon (green) and global reference frames are shown. Light blue circles highlight the nine corners tracked throughout the acquired video.

The validation approach for the *IMU* and *camera* classes consisted in acquiring electrically-synchronized motion signals from a real IMU, a real camera and a stereophotogrammetric system (Vicon 460) equipped with six infrared (IR) cameras running at 100 Hz. Four IR reflective markers were rigidly attached to the plastic box shown in Figure 4. An off-the-shelf Microsoft Webcam (VGA resolution, 30 Hz) and an Xsens Mtx unit (100-Hz sampling rate) equipped with a tri-axial gyroscope, a tri-axial accelerometer and a tri-axial magnetic sensor were mounted on the same plastic support. The housing holes on the plastic support hosting the markers and the IMU were milled with a computer numerical control (CNC) machine. The IMU and marker reference frame may be thus assumed to be aligned, and a good estimate of the IMU position in the marker reference frame was available. The 6 DOF rigid transformation between the camera and the IMU frames was estimated as proposed in [27]. Finally, the camera was calibrated using the MATLAB Camera Toolbox [22], and the estimated intrinsic parameters are reported in Table 1.

**Table 1:** Estimated intrinsic camera calibration parameters.

*f_x_* (pixel)	*f_y_* (pixel)	α (°)	*cc_x_* (pixel)	*cc_y_* (pixel)
670.24	665.54	0.00	332.95	237.40

During the experiment, the plastic box was moved by hand for about two minutes in front of a chessboard. The chessboard pattern (printed over an A3 paper sheet, with 2-cm squares) was a convenient choice to provide a set of corner points with known 3D coordinates in the global frame. As depicted in Figure 4, the global frame was placed on the chessboard with the *y*-*z* plane containing the grid pattern. Therefore, all 3D features had an *x* coordinate equal to zero, whereas the *y* and *z* coordinates were calculated by knowing the square size of the chessboard pattern. The nine chessboard corners highlighted in Figure 4 were tracked along the entire video sequence with the pyramidal implementation of the Lucas–Kanade tracker (KLT) proposed in [28].

#### 2.3.1. Simulation Environment Validation

*IMU* and *camera* instances were created to simulate the experiment described above. Ground-truth positions and orientations from the stereo-photogrammetric system were used as input signals for the simulated instances. The known 3D chessboard corners coordinates in {n} were assigned to the *environment* instance to create a visual scene to be projected by the *camera* object. The Earth’s magnetic and gravitational fields were measured by the IMU itself at the beginning of the experiment. The obtained values were properly rotated in {n} and used for defining the reference vector fields in the *environment*. The parameters reported in Table 1 were used to construct the virtual camera instance.

The root mean square error (*RMSE*) and the correlation (*R* value) between real and simulated sensor signals were used to assess the capability of accurately reproducing real sensor output when no disturbances were applied.

#### 2.3.2. Case Study: EKF Consistency

The EKF described in Section 2.2 was tested in both the simulated and the measured scenarios. In each case, the filter output was compared to the ground-truth orientation from the Vicon system. The obtained errors were then compared to the respective estimated covariances returned by the filter to check the convergence and consistency of the EKF.

#### 2.3.3. Simulated Distribution of Magnetic Disturbances

The spatial distribution of the magnetic disturbances may be simulated by means of the *dipole* class. In order to provide the user with a typical example, 500 *dipole* instances were randomly distributed within a simulated indoor environment (an 8 × 10 m room) with a random orientation and a magnetization equal to 5 A·m^2^.

## 3. Results

### 3.1. Simulation Results

**Figure 5:**
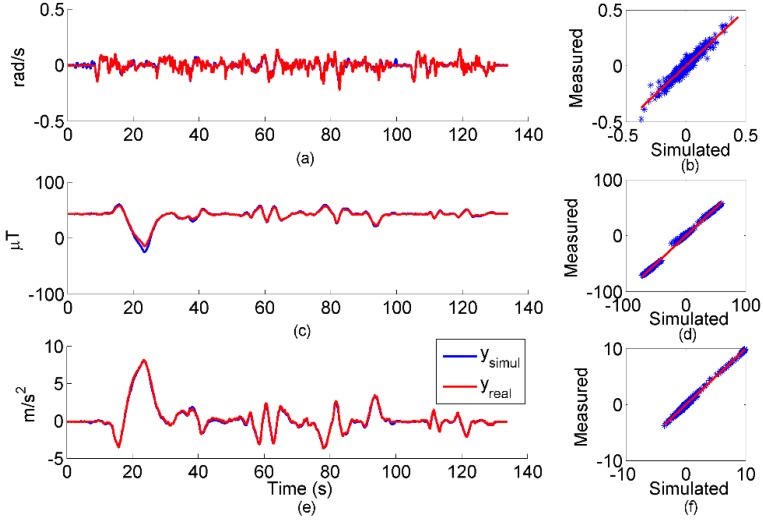
Comparison between simulated and measured IMU data: (**a**) y-axis gyroscope data; (**b**) three-axis scatter plot for gyroscope; (**c**) y-axis magnetometer data; (**d**) three-axis scatter plot for magnetometer; (**e**) y-axis accelerometer data; (**f**) three-axis scatter plot for accelerometer.

To validate the *IMU* and *camera* classes, the sensor outputs simulated in ideal conditions (*i.e.*, without calibration errors, magnetic disturbances, *etc.*) were compared to real measurements. Typical white measurement noises were considered for each sensor to prepare the simulated data for the EKF. In Figure 5, simulated and real signals acquired during the experiment described in Section 2.3 are shown.

For the sake of conciseness, only the y-axis data are reported. Scatter plots of all data are also presented, whereas *RMSE* and *R* correlation coefficients are reported in Table 2.

**Table 2:** *RMSE* and *R* between real and simulated IMU signals.

	Gyroscope		
	x	y	z
*RMSE* (rad/s)	0.03	0.02	0.02
*R*	0.97	0.90	0.97
	Magnetic Sensor		
*RMSE* (µT)	1.74	2.00	1.10
*R*	0.98	0.99	0.99
	Accelerometer		
*RMSE* (m/s^2^)	0.15	0.15	0.15
*R*	0.98	0.99	0.99

The smaller *R* value (0.90) was observed for the gyroscope about the y-axis (reported in Figure 5).

**Figure 6:**
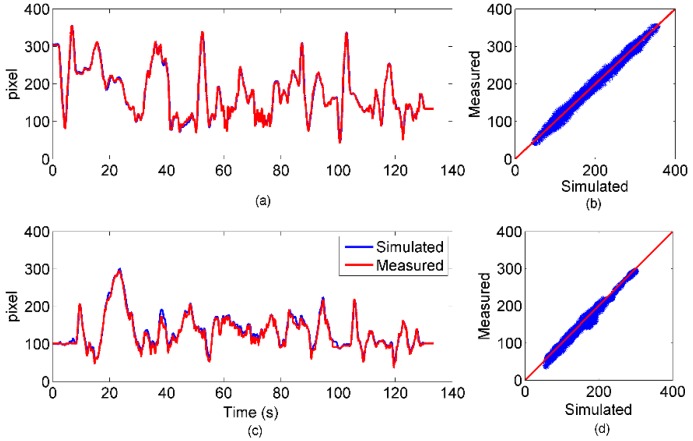
Comparison between simulated and measured camera output for one tracked feature: (**a**) x-axis comparison; (**b**) scatter plot for the x-axis feature data; (**c**) y-axis comparison; (**d**) scatter plot for-axis feature data.

The features simulated by the ideal camera were compared to their counterparts measured by the KLT. For clarity, the trajectories and the scatter plots shown in Figure 6 refer to only one of the nine tracked features.

The *RMSE* and *R* values were calculated for each of the nine features. The mean and standard deviations are reported in Table 3.

**Table 3:** Mean and standard deviations of *RMSE* and *R* values obtained comparing real and simulated 2D image features.

	x	y
***RMSE* (pixel)**	7.62 (0.70)	9.27 (0.99)
***R***	0.99 (0.00)	0.98 (0.00)

### 3.2. EKF Results: Orientation Estimation and Filter Consistency

In Table 4, the *RMSEs* obtained in both the simulated and the real scenarios are reported for the three DOFs (yaw, pitch and roll angles).

**Table 4:** *RMSE* obtained in both the simulated and real scenario for the three estimated Euler angles.

	*RMSE* Yaw (°)	*RMSE* Pitch (°)	*RMSE* Roll (°)
**Simulation**	0.11	0.10	0.12
**Real data**	0.53	0.63	0.96

**Figure 7:**
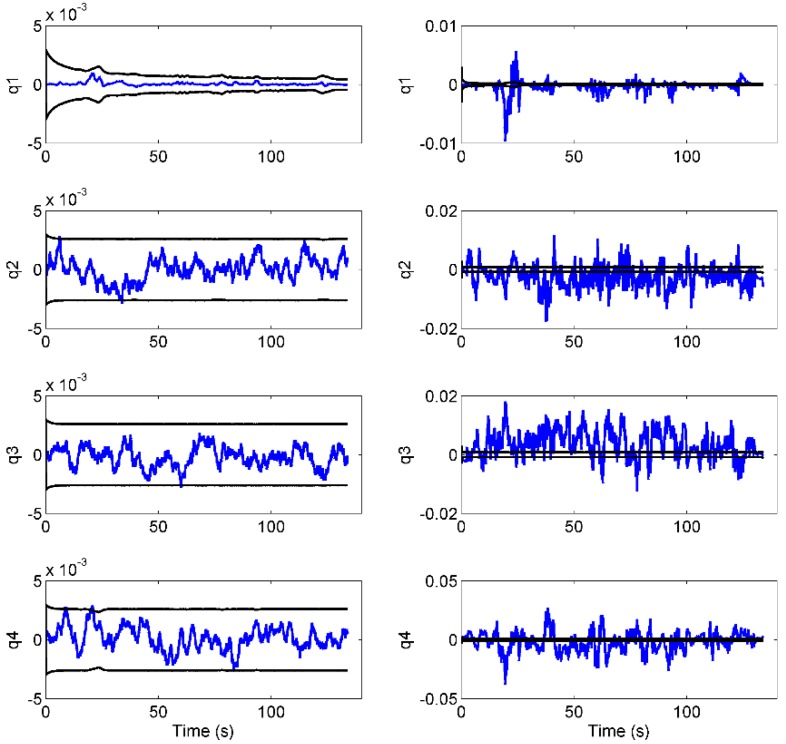
Estimation error trends (in blue) obtained for the four quaternion components in the simulated (**right**) and measured (**left**) scenarios; in black, the 99% confidence intervals.

As expected, when the EKF was provided with simulated data, considerably smaller errors were produced with respect to the real data case. In addition, the trends of the estimation errors relative to the four quaternion components and the relative estimated uncertainty are shown in Figure 7, for both scenarios.

### 3.3. Indoor Magnetic Disturbance Simulation

**Figure 8:**
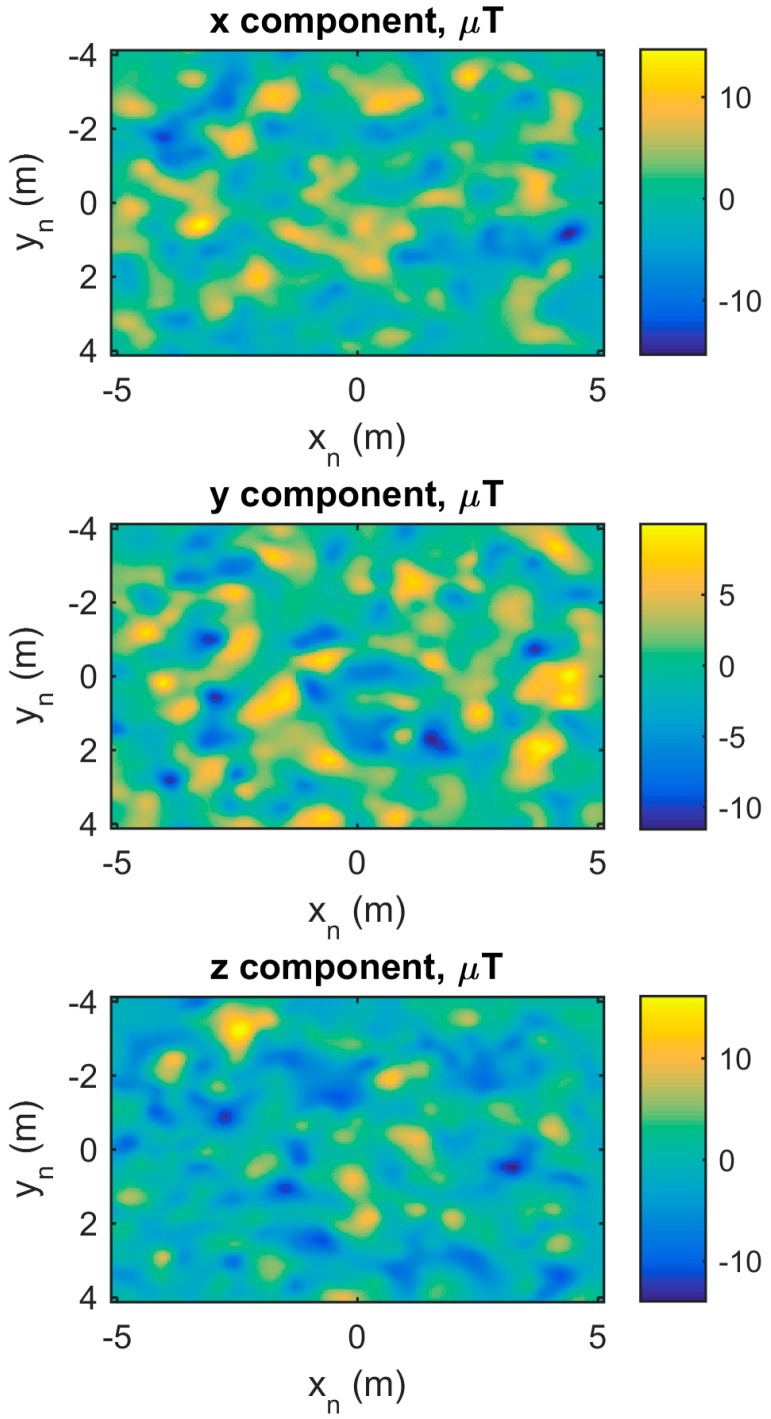
Spatial distribution of the magnetic disturbance within a simulated indoor environment.

The spatial distribution of the magnetic disturbances obtained from the simulation with 500 dipoles is depicted in Figure 8. The three components of the magnetic field are reported as a function of the *x* and *y* spatial directions, whereas the height (*z* direction) is kept constant.

## 4. Discussions

On the whole, the results shown in Section 3.1 demonstrated an overall agreement between the simulated and real sensor measurements. The *RMSE* values were about one tenth of the respective peak-to-peak magnitude measured both for the *IMU* and the *camera* classes. High correlations were achieved, as well, with a minimum value of 0.90 for the *y*-axis of the gyroscope. On that channel, the measurement noise of the real sensor was not negligible with respect to the strength of the measured signal. In particular, the results about the magnetometer are surprisingly good, because they were obtained without attempting any magnetic compensation. However, either enlarging the volume explored during the experiment or approaching a ferromagnetic object would have certainly degraded the correspondence between the simulated and real magnetometer data. In fact, as demonstrated in [20], uncompensated magnetic distortions can severely affect the results of the magnetic sensor simulation simply because the sensed magnetic field is not the Earth’s pure magnetic field. The *camera* instance also replicated the actual measurements accurately. The positive results prove the overall correctness of both the simulation environment implementation and the experimental design. In fact, *R* values were very close to one, and the *RMSEs* collected in about 140 s were lower than 10 pixel. Therefore, the proposed simulation framework proved to be a suitable tool for reproducing a real multi-sensor experiment.

To show one of the typical applications of the presented data simulator, we performed a consistency test on an EKF-based orientation estimator relying on both IMU and vision data. Referring to Table 4, the estimator proved to be accurate, returning errors of about 0.1° (on each Euler angle) in simulation and less than 1° with real data. However, we were interested in comparing the behavior of the EKF in the two scenarios, rather than the mere errors. In fact, the usefulness of the simulated study came out from the trends shown in Figure 7. The plot reported in Figure 7, for example, shows us that if we had not performed a simulated test, we could not have received any assurance about filter consistency. The real errors, *i.e.*, the errors computed with respect to the Vicon ground-truth data, are in fact considerably larger than the estimated covariances. This condition is usually interpreted as a sign of filter malfunctioning. However, by performing the same test with simulated data (from the same real experiment), we obtained the expected behavior, as shown in Figure 7. The errors remained within the estimated uncertainty during the entire trial, which means that the uncertainty was reliably estimated. Therefore, in a real case scenario, the inconsistency between the estimated covariances and the estimation errors (which are relatively large if compared to those obtained with simulated data) should be attributed to the slight imperfections of the experimental setup (misalignments, imperfect calibrations, *etc.*), rather than to filter instability. In fact, it should be noted that, for our setup, we estimated that the stereophotogrammetric errors propagated to the angles of interest in this study, causing a maximal inaccuracy of 0.5°. The EKF code therefore can be considered reasonably reliable, as the overall filter structure.

Finally, the proposed simulator allows the simulation of spatial magnetic disturbance distributions as the ones shown in Figure 8. This functionality has several practical applications, e.g., evaluating the orientation estimator robustness with respect to the magnetic disturbances or benchmarking the localization methods based on spatial magnetic anomalies [29,30].

## 5. Conclusions

In this paper, a data simulator for sensor fusion pose estimator benchmarking was presented. The plausibility of the simulated outputs was numerically assessed through comparisons with real sensors. In addition, an example of quaternion-based EKF benchmarking was shown in order to demonstrate the usefulness of the proposed simulation framework. The code relative to the proposed simulator is attached to the present paper as Supplementary Material.

## Supplementary Files

Supplementary File 1
