# Supplementary material for: A Simulation Environment for Benchmarking Sensor Fusion-Based Pose Estimators
Source: Sensors (Basel). 2015 Dec 19;15(12):32031–44. doi: 10.3390/s151229903 (PMC4721821; doi:10.3390/s151229903)

Demo 1: Simulated experiment

1. Add the folders "commonFunctionsAndData" and "SimulatorCode" to the path (with subfolders);
2. Set the folder "SimulatedExperiment" as the current directory;
3. Run "TrackingTest.m";
4. After a while a similar screenshot will appear:

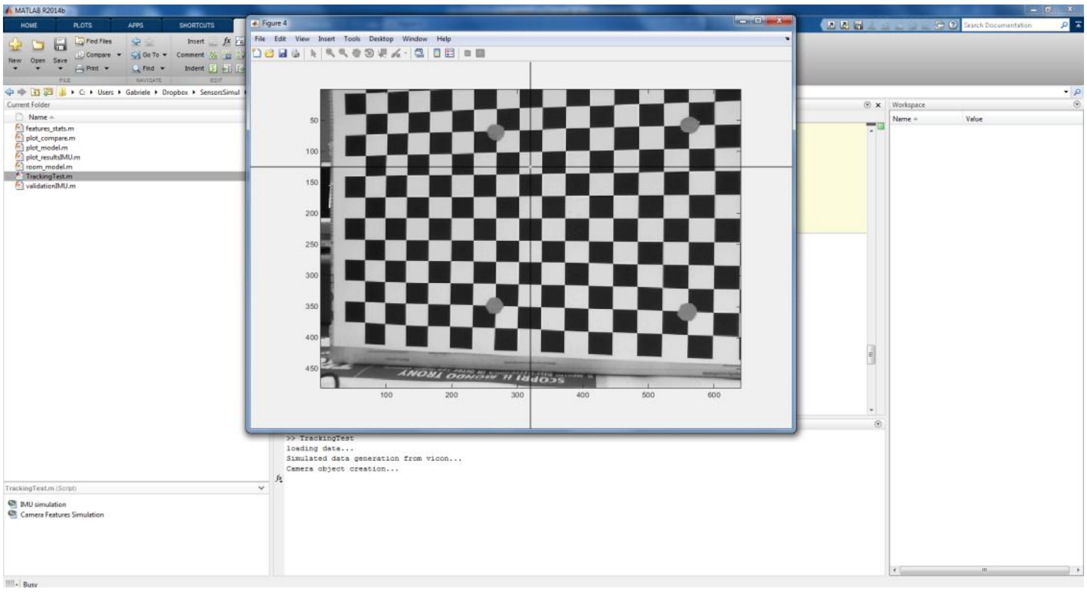

5. Select the four corners shown below (counterclockwise) by means of the ginput tool, starting from the upper left one;

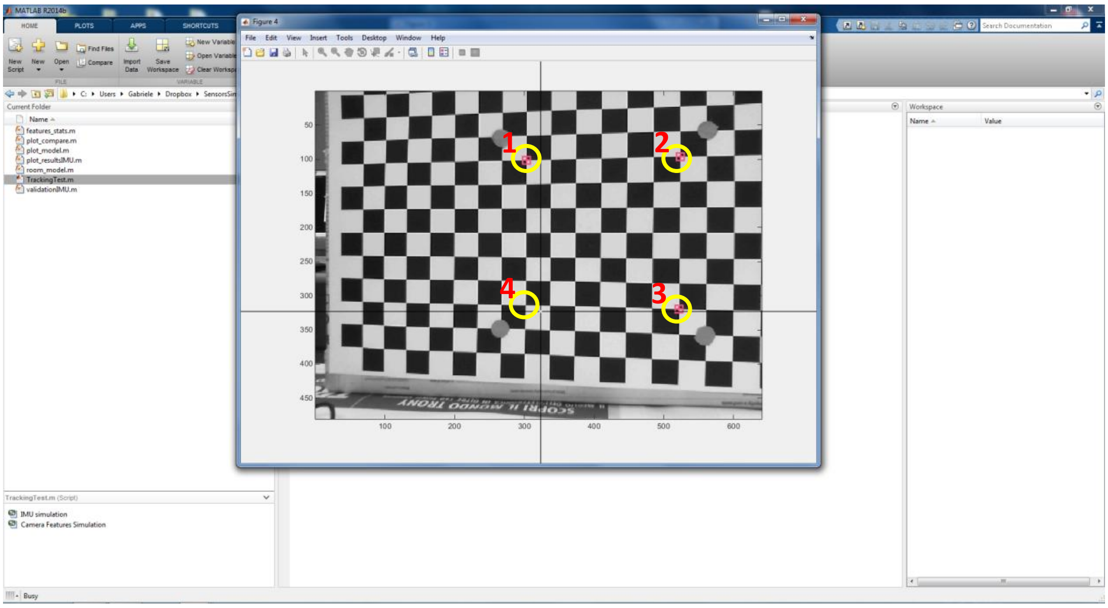

6. After the four corners are selected, the Lucas-Kanade tracker is launched.

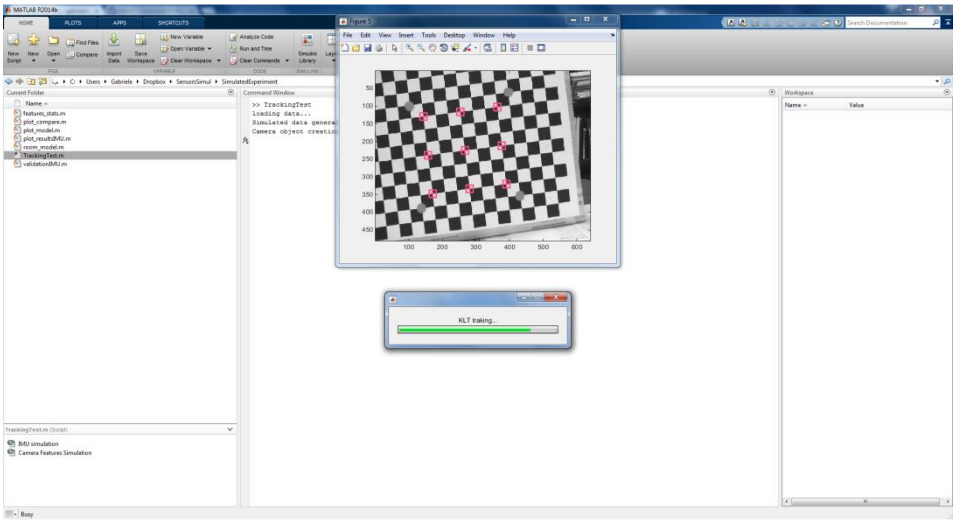

7. When the program ends all the simulated variables may be found in the workspace.

Demo 2: Magnetic Map

1. Add the folders "commonFunctionsAndData" and "SimulatorCode" to the path (with subfolders);
2. Set the folder "MagneticMap" as the current directory;
3. Run "MapEvaluation.m";
4. If the "Parallel Computing Toolbox" is installed, the execution will be parallelized.
5. When the program ends, the following magnetic maps will be shown.

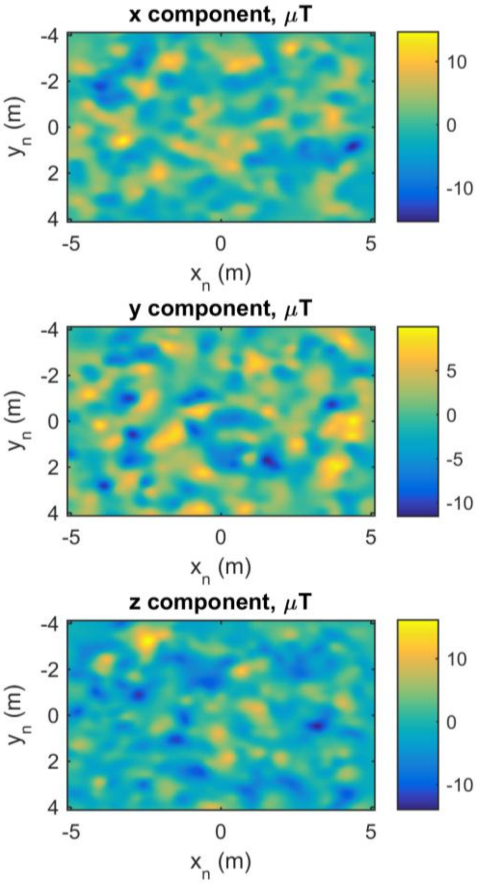

Supplement: Supplementary File 1 [file sensors-15-29903-s001.zip › SensorsSupplementaryMaterial/README.pdf]
